# Acute Macular Neuroretinopathy and Paracentral Acute Middle Maculopathy during SARS-CoV-2 Infection and Vaccination

**DOI:** 10.3390/vaccines11020474

**Published:** 2023-02-17

**Authors:** Parthopratim Dutta Majumder, Aniruddha Agarwal

**Affiliations:** 1 Medical Research Foundation, Sankara Nethralaya, 18, College Road, Chennai 600006, India; 2 Eye Institute, Cleveland Clinic Abu Dhabi (CCAD), Abu Dhabi P.O. Box 112412, United Arab Emirates; 3 Cleveland Clinic Lerner College of Medicine, Case Western Reserve University, Cleveland, OH 44195, USA; 4 Department of Ophthalmology, Maastricht University Medical Center, 6211 LK Maastricht, The Netherlands

**Keywords:** acute macular neuroretinopathy, paracentral acute middle maculopathy, SARS-CoV-2 infection, COVID-19, vaccination, optical coherence tomography

## Abstract

*Purpose:* To review the demographic and clinical profile of patients developing acute macular neuroretinopathy (AMN) or paracentral acute middle maculopathy (PAMM) after receiving coronavirus disease-2019 (COVID-19) vaccination or infection. *Methods:* In this review article, the published literature was searched to determine cases developing either AMN or PAMM after COVID-19 vaccinations or infections. Data, including demographic profile, presenting features, symptoms, diagnosis, and clinical outcomes, were extracted from the selected publications. These parameters were compared between the two groups, i.e., patients developing AMN/PAMM either after vaccination or infection. *Results:* After the literature review, 57 patients developing either AMN (*n* = 40), PAMM (*n* = 14), or both (*n* = 3) after COVID-19 infection (*n* = 29) or vaccination (*n* = 28) were included (mean age: 34.9 ± 14.4 years; *n* = 38; 66.7% females). In 24.6% patients, the diagnosis of COVID-19 infection was preceded by the development of ocular disease. There were no significant differences in the age or gender between the patients developing AMN or PAMM after vaccination or infection (*p* > 0.13). Among the vaccination group, the highest number of patients developing AMN/PAMM were after the Oxford-AstraZeneca (*n* = 12; 42.9%). Patients with vaccination had a significantly early onset of AMN/PAMM compared to those with infection (11.5 ± 17.6 days versus 37.8 ± 43.6 days; *p* = 0.001). *Conclusions:* Both AMN and PAMM are reported to be associated with COVID-19 infections and in persons receiving vaccination against COVID-19. While COVID-19 infections and vaccinations may have a contributory role, other risk factors such as oral contraceptive pills may also play a role in the development of the disease.

## 1. Introduction

Novel SARS-CoV-2 infection is attributed to the result of a hypercoagulable state, which can induce thrombus formation leading to local embolism in the small vessels and micro-vessels of the relevant target organs [1]. Various other pathological events leading to a pro-inflammatory and anti-fibrinolytic state, such as the development of antiphospholipid antibodies and increase in D-dimer levels, were reported to contribute to the hypercoagulable state in patients with COVID-19 [1]. Similarly, several reports of unusual thromboembolic adverse events were reported following the widespread use of anti-SARS-CoV-2 vaccinations. In accordance with the literature, various clinical conditions secondary to thromboembolic and ischemic episodes were reported in the eyes of patients with COVID-19 infection and in patients receiving anti-SARS-CoV-2 vaccinations [2,3].

In a prospective cross-sectional study by Sim et al., one in nine patients with COVID-19 infections had microvascular alterations on ocular imaging, and these signs were observed even in asymptomatic patients with normal vital signs [4]. On the other hand, the exact cause or association between such microvascular changes and COVID-19 vaccination is not clear, but there are several reports that have highlighted the role of vaccine-induced inflammation.

Acute macular neuroretinopathy (AMN) is a rare, outer retinal, microvascular disorder characterized by acute onset wedge-shaped or petaloid perifoveal lesions and ischemia of the deep retinal capillary plexus, involving the external limiting membrane and photoreceptors. Paracentral acute middle maculopathy (PAMM) is also a manifestation of outer retinal ischemia with intermediate/deep retinal capillary plexus involvement but limited to the inner nuclear layer, outer plexiform/nuclear layers. Various systemic risk factors are reported in association with AMN: flu-like illness, fever, oral contraceptive use, systemic shock, antecedent trauma, and dengue fever, among others. A non-specific flu-like illness or fever was reported in almost half of the patients in a review of 101 cases of AMN [5]. PAMM was reported with various other retinal vascular diseases such as retinal vascular occlusions, inflammatory chorioretinopathies, congenital glaucoma, foveal hypoplasia, various intraocular and extraocular surgeries, and systemic vascular diseases, as well as in healthy patients [6]. Recently, there has been an increase in the literature concerning these two entities in patients with SARS-CoV-2 infection and in patients with anti-SARS-CoV-2 vaccinations.

## 2. Materials and Methods

The study was designed as a review of published AMN and PAMM cases in the literature associated with COVID-19 infections or vaccination. Since the study analyzed published manuscripts, institutional ethics clearance was not required for the study, and there were no informed consent documents. The study adhered to the tenets of the declaration of Helsinki. We conducted a detailed review of the literature on Medline (National Library of Medicine/PUBMED) from December 2020 until 30 September 2022. The search was conducted by two ophthalmologists (retina specialists with fellowship training) using terms such as ‘acute macular neuroretinopathy’, ‘paracentral acute middle maculopathy’ AND/OR ‘SARS-CoV-2′, ‘COVID-19’, and ‘COVID-19 vaccine’. The bibliographies of the retrieved articles were searched thoroughly to find relevant articles.

The manuscripts in the English language were selected for further analysis only. The inclusion criteria were published cases, case series, or original articles of patients with the diagnosis of either AMN, PAMM, or both associated with COVID-19 infection or vaccination. We included studies with either definitive diagnosis of COVID-19 or recipients of COVID vaccination (any approved type of vaccine), including booster doses. Cases without a definitive diagnosis of COVID-19 infection were excluded from this analysis [7]. If a case exhibited pre-existing AMN or PAMM, they were excluded from the analysis. The definition of AMN and PAMM was used based on the published manuscripts, essentially with the help of clinical examination, fundus photography, and multimodal imaging techniques including optical coherence tomography (OCT), fluorescein angiography (FA), and optical coherence tomography angiography (OCTA), whichever were available.

For the purpose of the study, we collected available demographic data from the published cases, including data such as gender, age, ethnicity, and geographic location (if available). The data concerning previous or current COVID-19 infection and vaccination (type, number of doses, and time since last dose) were noted. The medical history of the patients available in the published cases, such as history of drug intake, pre-existing medical conditions, previous surgeries, or ocular findings were noted. The details of the ophthalmic examination and imaging findings were noted for each case. The course of the AMN and PAMM lesions were obtained, and treatments prescribed were also noted. Any additional clinical data such as additional diagnoses, complications, or findings were noted.

The data were collected in a pre-designed data collection sheet by two ophthalmologists. Data analysis was performed using GraphPad Prism^®^ (GraphPad Software Inc., La Jolla, CA, USA) version 6.0. The demographic data were expressed in mean and standard deviation. The parameters between two groups, namely, AMN versus PAMM, and AMN or PAMM after vaccination or after COVID-19 infection were compared using Mann–Whitney U test. Binary data between the groups were analyzed using Chi Square test. The clinical findings, course of the disease, complications and other data were described using descriptive statistics. The total follow-up was also described using mean and standard deviation. The statistical significance was denoted by *p* < 0.05 and 95% confidence interval.

## 3. Results

A total of 57 cases of AMN and PAMM were obtained after analysis of the published literature. The mean age of all the patients included in the analysis was 34.9 ± 14.4 years. Among them, 29 developed either AMN or PAMM following COVID-19 infections (50.9%) (Table 1), and the other 28 had a history of recent COVID vaccination (49.1%). There were 38 females in the cohort (66.7%). (Table 1 and Table 2 describe the demographic features of the patients included in the study). The number of reported cases of AMN (*n* = 40; 70.2%) was higher than PAMM (*n* = 14; 24.6%). PAMM was more commonly seen in patients with COVID-19 infections (*n* = 10) than in patients following vaccinations (*n* = 4). PAMM was associated with central retinal artery occlusion (CRAO) in two patients with COVID-19 infection and in one patient with cilioretinal artery occlusion [8,9,10]. Turedi and Onal Gunay described the case of a 54-year-old male who developed CRAO two weeks following COVID-19 infection [8]. The laboratory work-up was non-contributory, and ancillary imaging after five days of diagnosis of CRAO revealed a diagnosis of coexisting PAMM [8]. In another case report by Matilde et al., a 41-year-old male presented with an optic neuritis-like picture in the left eye and was treated with pulse corticosteroid therapy. After three days, the patient developed Purtscher-like retinopathy, and a diagnosis of atypical CRAO was considered. OCT of the left eye revealed a PAMM lesion in this patient [9]. Subsequently, the patient tested positive at PCR for COVID-19 infection after he developed flu-like symptoms [9]. Two patients had combined features of AMN and PAMM in patients with the COVID-19 infected group, whereas one such case was reported in a vaccinated patient [11,12,13].

In 14 patients (24.6%), the diagnosis or confirmation of COVID-19 infection was preceded by the ocular symptoms and/or signs of AMN/PAMM [11,14,15]. Bilateral involvement was almost similar in both cohorts. The patients who developed AMN and PAMM after COVID-19 vaccinations were relatively younger than the cohort with COVID-19 infection, though it did not reach statistical significance (32.04 ± 13.2 years versus 36.7 ± 14.4 years; *p* = 0.13). Female predilection was more frequently observed in the cohort with COVID vaccination (though not statistically significant) (21 out of 28 patients; 75% versus 18 out of 29 patients; 62%) (*p* = 0.29). Among the female patients, 71% were either on or were using oral contraceptive pills or hormonal devices for birth control.

A total of 21 patients developed AMN and PAMM after the first dose of vaccination, Oxford-AstraZeneca (ChAdOx1-S (recombinant) vaccine) (*n* = 12) was the most common vaccine received by the patients in this group, followed by BNT162b2 (Pfizer Inc./BioNTech SE, Mainz, Germany) (referred to as Pfizer-BioNTech vaccine) and Sinopharm BIBP COVID-19 vaccine (referred to as Sinopharm BIBP vaccine) (*n* = 5 each), mRNA-1273 (Moderna Therapeutics Inc., Cambridge, MA, USA) (referred to as Moderna vaccine) (*n* = 4). One patient developed AMN in both eyes after Ad26.COV2.S (Janssen Pharmaceuticals, Beerse, Belgium) (referred to as Janssen Vaccine). Fourteen patients exhibited bilateral involvement in the vaccinated group, and among them, 12 had AMN and in one patient, both AMN and PAMM were described after COVID-19 vaccination. The details are provided in Table 2.

The cohort of patients with COVID-19 vaccination exhibited significantly early onset of AMN and PAMM when compared with the patients with SARS-CoV-2 infection (11.5 ± 17.6 days versus 37.8 ± 43.6 days; *p* = 0.001). One patient developed ocular symptoms within 20 min of vaccination, along with systemic symptoms such as malignant hypertension [33].

## 4. Discussion

Since its first description in 1975, one systemic review identified 101 cases of reports of AMN published before December 2014 [5] Our literature review shows a tremendous increase in reports of AMN and PAMM following COVID-19 infection and COVID-19 vaccinations in comparison to the previously published literature on these two clinical entities. The increase in awareness of these clinical entities and the greater use of optical coherence tomography (OCT) and other imaging such as OCT angiography (OCTA) in clinical practice are probably responsible for such an increase in these reports. Furthermore, viral flu-like illness and fever are already known systemic risk factors for AMN. In the published literature, we found a higher proportion of female patients developing AMN and PAMM. This could be attributed to a high percentage (more than 70%) of the female patients using hormone control and OCP that may have contributed to the increased risk of thromboembolic events in patients receiving COVID-19 vaccines. Therefore, due to the presence of confounding factors, our findings do not indicate that females may be more prone to developing retinal ischemic events following COVID-19 infections or vaccinations. This needs further assessment in the future studies.

It may be possible that many cases of AMN and PAMM resulting due to COVID-19 infections or vaccinations may be under-reported. There are other publicly available databases that provide information following adverse events related to vaccination, notably, the Vaccine Adverse Event Reporting System (VAERS), which is co-sponsored by the Centers of Disease Control (CDC) and the United States Food and Drug Administration (US-FDA). The VAERS is a self-reporting system available to the general population, as well as physicians, who can enter and report adverse events related to their vaccination. Since this is a freely available resource and voluntary in nature, it does not capture all the details regarding the adverse event, and there could be events that are unreported in VAERS database. In our study, we have not performed a VAERS database search. This is because data from VAERS are not peer-reviewed, and therefore, it is inappropriate to combine it with the data obtained from published research for this manuscript. The disease manifestations of AMN and PAMM may also be very subtle in nature. Considering the self-limiting course of these two conditions and possible minimal clinical signs and symptoms, many such cases of AMN/PAMM may have been unnoticed and, hence, were not reported, leading to a reporting bias.

A significant risk of systemic thrombotic complications leading to complement-mediated thrombotic microangiopathy in SARS-CoV-2 infection is now well proven. Our review showed a higher number of PAMM cases in patients with COVID-19 infection. Patients with COVID-19 infection are, thus, at higher risk of developing the acute phase of ischemia of retinal capillary plexuses that may lead to PAMM. To add to it further, PAMM was reported in patients with pulmonary embolism [16] in association with retinal artery occlusions [8,10,12] and raised D-dimer levels [41].

Our study has various limitations including reporting bias, as stated earlier. Other limitations include lack of uniform diagnostic testing and follow-up and lack of treatment guidelines or uniformity. There may be cases where the cause-and-effect association may be weak. It is possible that there could be multiple confounding factors that could exist, leading to the development of AMN and PAMM. Due to the significant interest in the ocular and systemic effects of COVID-19 in the scientific community, it is possible that there could be a publication bias leading to a higher number of reports on AMN and PAMM following either COVID-19 infection or vaccination. The pathophysiological mechanisms of the development of deep retinal ischemia such as AMN or PAMM after vaccination (including vaccines with different mechanisms of action) and COVID-19 infection are still speculative and under investigation. Based on the available reports, it is difficult to conclude which vaccine confers a higher risk of either AMN or PAMM in the recipients.

## 5. Conclusions and Future Directions

In summary, both COVID-19 infection and vaccination against COVID-19 have been implicated with the development of AMN and PAMM detected using clinical examination and multimodal imaging using OCT and OCTA. The exact strength of this association, or the level of contribution of COVID-19 vaccine/infection in comparison with other risk factors such as OCP, is yet to be ascertained. Patients developing AMN or PAMM after COVID-19 vaccination tend to be younger and develop the disease within a shorter duration of time compared to those developing the disease after COVID-19 infection.

In the future, it is necessary to focus research on the true risk of ischemic events arising from both COVID-19 infections and vaccinations and if there are certain exogenous or endogenous risk factors that contribute to a higher risk of thromboembolic events compared to the general population. With a hope that the future burden of COVID-19 disease will reduce, it will become imperative to understand the real safety and efficacy of the vaccination itself and if there could be improvements in the molecular composition that may reduce adverse events attributed to the vaccine.

## Figures and Tables

**Table 1:** Review of Literature of AMN and PAMM cases in COVID-19 patients.

Author	Age/Sex	Duration (Days)	Laterality	Diagnosis	Presenting Symptoms	Clinical Findings	Outcome
Azar et al. [7]	21/M	NA	Right Eye	AMN	Central Scotoma	NA	Favorable
	28/F	NA	Both Eyes	AMN	Paracentral scotoma	NA	NA
	27/F	NA	Both Eyes	AMN	Paracentral scotoma	NA	NA
	22/F	NA	Right Eye	AMN	Paracentral scotoma	NA	NA
Turedi and Onal Gunay [8]	54/M	14	Right Eye	CRAO + PAMM	Vision loss	Pale, white retina and “cherry-red spot appearance”	
Matilde et al. [9]	41/M	-	Left Eye	atypical CRAO, PAMM	Decreased vision	Initially unremarkable, multiple Purtscher-like CWS, slight retinal whitening around the fovea, and cherry-red spot	Favorable
Ozsaygılı et al. [10]	26/F	14	Left Eye	PAMM	Central Visual Field Defect	Focal area of well-demarcated retinal whitening over the distribution of a CILRA in the superior papillomacular bundle region	Favorable
Gascon et al. [11]	53/M	-	Left Eye	AMN/PAMM	Loss of vision, Negative scotoma, Dyschromatopsia.	Retinal hemorrhages, Roth spots, subtle whitish Parafoveal lesions.	NA
Goyal et al. [12]	32/M	120	Both Eyes	AMN/PAMM	Paracentral and triangular negative scotoma	Triangular deeper retinal greyish-white lesion located superonasal to center of macula in right eye	NA
Preti et al. [14]	70/M	-	Left Eye	AMN	Diaphoresis, Paracentral scotoma, Vision loss	OD: Unremarkable OS: Old scleral buckle	Favorable
Capuano et al. [15]	27/M	-	Left Eye	PAMM	Paracentral scotoma, Dyschromatopsia	Subtle yellowish perifoveal halo	Partial Improvement
	37/F	-	Both Eyes	AMN	Paracentral scotoma	Alternated foveal reflex	Favorable
Naughton et al. [16]	28/M	NA	Both Eyes	PAMM	Decreased vision	CWS, Intraretinal haemorrhages, Retinal pallor in fovea	Favorable
Mace and Pipelet [17]	39/F	2	Both Eyes	AMN	Photopsia, Para-central scotoma	Unremarkable	Persistent
Aidar et al. [18]	71/F	14	Left Eye	AMN	Diminution of vision	Foveal pigment mobilization	No Improvement
Padhy et al. [19]	19/F	14	Both Eyes	PAMM	Scotoma	CWS, subtle white lesions at macula	Favorable
Castro et al. [20]	36/F	63	Both Eyes	PAMM	Blurred vision	Superficial hemorrhages in macula and peripheral retina in left eye	Favorable
Jonathan et al. [21]	47/M	60	Right Eye	PAMM	Paracentral scotoma	Retinal Whitening	Scotoma persisted
Diafas et al. [22]	59/M	14	Both Eyes	AMN	Blurred vision	Unremarkable	Favorable
	24/F	7	Both Eyes	AMN	Paracentral Scotoma.	Perifoveal dark grey patches	No Improvement
Virgo et al. [23]	32/M	16	Right Eye	AMN	Paracentral Scotoma	Unremarkable	
	37/F	35	Left Eye	PAMM	Paracentral Scotoma.	Unremarkable	NA
Sonmez et al. [24]	41/F	30	Right Eye	PAMM	Paracentral Scotoma, Decreased Vision	Parafoveal hyper-pigmented round lesion and increased vascular tortuosity	NA
Giacuzzo et al. [25]	23/F	-	Both Eyes	AMN	Photopsias, Paracentral Scotomas	Unremarkable	Favorable
Jalink and Bronkhor [26]	29/F	150	Left Eye	AMN	Paracentral scotoma	Subtle alterations around fovea	Scotoma Unchanged
Zamani et al. [27]	35/F	-	Both Eyes	AMN	Paracentral scotoma, Photopsia	Multiple hemorrhages with white or pale center (Roth’s spots)	Died due to pneumonia
David and Fivgas [28]	22/F	-	Both Eyes	AMN	Scotoma	Multiple subtle reddish-brown petaloid lesions radiating from the fovea	NA
Masjedi et al. [29]	29/F	14	Left Eye	AMN	Paracentral scotoma	Unremarkable	Scotoma persisted
Deshmukh et al. [30]	56/F	-	Left Eye	AMN	Paracentral scotoma	Parafoveal retinal whitening	Stable

M = male, F = female, AMN = acute macular neuroretinopathy, PAMM = paracentral acute middle maculopathy, CRAO = central retinal artery occlusion, CWS = cotton wool spot.

**Table 2:** Review of Literature of Cases with AMN and PAMM cases in patients following COVID-19 vaccinations.

Authors	Age/Sex	Duration (Days)	COVID-19 Vaccine (Dose)	Diagnosis	Laterality	Presenting Symptoms	Clinical Features	Outcome
Vinzamuri et al. [13]	35/M	30	Oxford–AstraZeneca (2)	PAMM/AMN	Both Eyes	Blurred vision, Black spots	Unremarkable	Favorable
Diafas et al. [22]	54/M	21	Pfizer–BioNTech (1)	AMN	Left Eye	Photopsia, Small scotoma	Orange–brown oval-shaped lesion superotemporal to the fovea	NA
Jalink and Bronkhorst [26]	42/F	45	Moderna (2)	AMN	Right Eye	Paracentral Scotoma	Faint, brownish circle nasal to the fovea	Favorable
Zaheer et al. [31]	22/F	5	Oxford–AstraZeneca (1)	AMN	Right Eye	Two paracentral scotomas, Flashes	Unremarkable	Stable
Afonso et al [32]	28/F	2	Oxford–AstraZeneca (1)	AMN	Both Eyes	Paracentral Scotoma	Red brown petaloid lesions around the fovea.	Slight improvement
Pichi et al. [33]	NA	5	Sinopharm BIBP (1)	AMN	Left Eye	Vision Loss	NA	Favorable
	NA	NA	Sinopharm BIBP (1)	AMN	NA	NA	NA	
	NA	1 ^#^	Sinopharm BIBP (1)	PAMM	Left Eye	Blurring of vision, Inferior scotoma	A dot hemorrhage superior to the fovea.	NA
Patel and Yonekawa [34]	26/F	2	Janssen (1)	AMN	Both Eyes	Paracentral Scotoma	Unremarkable	NA
Bohler et al. [35]	27/F	2	Oxford–AstraZeneca (1)	AMN	Left Eye	Paracentral Scotoma	Teardrop-shaped macular lesion	NA
Book et al. [36]	21/F	3	Oxford–AstraZeneca (1)	AMN	Both Eyes	Paracentral Scotoma	Circumscribed paracentral dark lesions.	NA
Dehghani et al. [37]	38/M	14	Sinopharm BIBP (1)	PAMM	Right Eye	Flashes of light, Scotoma	Unremarkable	NA
Mambretti et al. [38]	22/F	2	Oxford–AstraZeneca (1)	AMN	Right Eye	Scotoma	Unremarkable	NA
	28/F	2	Oxford–AstraZeneca (1)	AMN	Right Eye	Paracentral scotoma	Unremarkable	NA
Bolletta et al. [39]	24/F	2	Oxford–AstraZeneca (1)	AMN	Both Eyes	Visual field defect	NA	Favorable
Ishibashi et al. [40]	33/F	8	Pfizer–BioNTech (2)	AMN	Left Eye	Field defect	Unremarkable	NA
	62/M	7	Pfizer–BioNTech (2)	PAMM	Left Eye	Visual field defect	Unremarkable	NA
Malerbi et al [41]	50+/F	30	Sinopharm BIBP (1)	PAMM	Both Eyes	Scotoma	Subtle white lesions at the macula in RE and marked lesions in LE	Favorable
Sanjay S et al. [42]	25/F	3	Oxford–AstraZeneca (1)	AMN	Both Eyes	Diminution of Vision, Scotoma	Unremarkable	NA
Drüke D et al. [43]	23/F	1	Oxford–AstraZeneca (1)	AMN	Both Eyes	Paracentral Scotoma	a subtle brownish rimmed lesion parafoveal in the right eye and a bigger blurred lesion nasal to the macula in the left eye	Favorable
Valenzuela DA et al. [44]	20/F	2	Pfizer–BioNTech (2)	AMN	Both Eyes	Photopsias, Scotomata	Unremarkable	Favorable
Rennie AT et al. [45]	21/F	3	Moderna (2)	AMN	Both Eyes	Paracentral Scotomas	Perifoveal intraretinal hemorrhage, Reddish-brown perifoveal lesions	Favorable
Bellur S et al. [46]	64/F	3	Moderna (1)	AMN	Both Eyes	Diminution of Vision	Subtle, pigmentary changes in Macula	Mild Improvement
Franchi A et al. [47]	19/F	1	Oxford–AstraZeneca (1)	AMN	Both Eyes	Sudden onset of fortifications	Large, opaque-appearing parafoveal wedge-shaped areas	Favorable
	31/F	2	Oxford–AstraZeneca (1)	AMN	Both Eyes	Sudden onset of fortifications, Paracentral Scotoma	Small opaque-appearing area superior to the fovea	Favorable
	40/F	45	Moderna (2)	AMN	Right Eye	Blurred vision, Photopsia	Pigmentary Changes	Favorable
Gabrielle PH et al. [48]	25/F	1	Oxford–AstraZeneca (1)	AMN	Both Eyes	Paracentral Scotoma, Blackspots	Unremarkable	NA
Chen S and Hodge C [49]	21/F	70	Pfizer–BioNTech (1)	AMN	Left Eye	Paracentral Scotoma	Oval parafoveal lesions	NA

M = male, F = female, AMN = acute macular neuroretinopathy, PAMM = paracentral acute middle maculopathy, ^#^ Symptoms started within 20 min of vaccination.

## Data Availability

Not applicable.

## References

[1] Kichloo A., Dettloff K., Aljadah M., Albosta M., Jamal S., Singh J., Wani F., Kumar A., Vallabhaneni S., Khan M.Z. (2020). COVID-19 and Hypercoagulability: A Review. Clin. Appl. Thromb..

[2] Sen S., Kannan N.B., Kumar J., Rajan R.P., Kumar K., Baliga G., Reddy H., Upadhyay A., Ramasamy K. (2022). Retinal manifestations in patients with SARS-CoV-2 infection and pathogenetic implications: A systematic review. Int. Ophthalmol..

[3] Dutta Majumder P., Prakash V.J. (2022). Retinal venous occlusion following COVID-19 vaccination: Report of a case after third dose and review of the literature. Indian J. Ophthalmol..

[4] Sim R., Cheung G., Ting D., Wong E., Wong T.Y., Yeo I., Wong C.W. (2022). Retinal microvascular signs in COVID-19. Br. J. Ophthalmol..

[5] Bhavsar K.V., Lin S., Rahimy E., Joseph A., Freund K.B., Sarraf D., Cunningham E.T. (2016). Acute macular neuroretinopathy: A comprehensive review of the literature. Surv. Ophthalmol..

[6] Moura-Coelho N., Gaspar T., Ferreira J.T., Dutra-Medeiros M., Cunha J.P. (2020). Paracentral acute middle maculopathy-review of the literature. Graefes Arch. Clin. Exp. Ophthalmol..

[7] Azar G., Bonnin S., Vasseur V., Faure C., Salviat F., Clermont C.V., Titah C., Farès S., Boulanger E., Derrien S. (2021). Did the COVID-19 Pandemic Increase the Incidence of Acute Macular Neuroretinopathy?. J. Clin. Med..

[8] Turedi N., Onal Gunay B. (2022). Paracentral acute middle maculopathy in the setting of central retinal artery occlusion following COVID-19 diagnosis. Eur. J. Ophthalmol..

[9] Matilde R., Alberto P., Fabio G., Leonardo T., di Geronimo N., Michela F., Costantino S. (2022). Multitarget microangiopathy in a young healthy man with COVID-19 disease: A case report. Indian J. Ophthalmol..

[10] Ozsaygılı C., Bayram N., Ozdemir H. (2021). Cilioretinal artery occlusion with paracentral acute middle maculopathy associated with COVID-19. Indian J. Ophthalmol..

[11] Gascon P., Briantais A., Bertrand E., Ramtohul P., Comet A., Beylerian M., Sauvan L., Swiader L., Durand J.M., Denis D. (2020). COVID-19-Associated Retinopathy: A Case Report. Ocul. Immunol. Inflamm..

[12] Goyal M., Murthy S., Annum S. (2021). Retinal Manifestations in Patients Following COVID-19 Infection: A Consecutive Case Series. Indian J. Ophthalmol..

[13] Vinzamuri S., Pradeep T.G., Kotian R. (2021). Bilateral paracentral acute middle maculopathy and acute macular neuroretinopathy following COVID-19 vaccination. Indian J. Ophthalmol..

[14] Preti R.C., Zacharias L.C., Cunha L.P., Monteiro M.L.R. (2022). Acute macular neuroretinopathy as the presenting manifestation of covid-19 infection. Retin. Cases Brief Rep..

[15] Capuano V., Forte P., Sacconi R., Miere A., Mehanna C.-J., Barone C., Bandello F., Souied E.H., Querques G. (2022). Acute macular neuroretinopathy as the first stage of SARS-CoV-2 infection. Eur. J. Ophthalmol..

[16] Naughton A., Ong A.Y., Gkika T., Downes S. (2021). Bilateral paracentral acute middle maculopathy in a SARS-CoV-2-positive patient. Postgrad. Med. J..

[17] Macé T., Pipelart V. (2021). Acute macular neuroretinopathy and SARS-CoV-2 infection: Case report. J. Fr. Ophtalmol..

[18] Aidar M.N., Gomes T.M., de Almeida M.Z.H., de Andrade E.P., Serracarbassa P.D. (2021). Low Visual Acuity Due to Acute Macular Neuroretinopathy Associated with COVID-19: A Case Report. Am. J. Case Rep..

[19] Padhy S.K., Dcruz R.P., Kelgaonkar A. (2021). Paracentral acute middle maculopathy following SARS-CoV-2 infection: The D-dimer hypothesis. BMJ Case Rep..

[20] Castro C.S., Ferreira A.S., Silva N.P., Lume M.R., Furtado M.J. (2022). Paracentral Acute Middle Maculopathy After COVID-19 Disease: Multimodal Evaluation. Retin. Cases Brief Rep..

[21] Jonathan G.L., Scott F.M., Matthew K.D. (2021). A Case of Post-COVID-19-Associated Paracentral Acute Middle Maculopathy and Giant Cell Arteritis-Like Vasculitis. J. Neuro-Ophthalmol..

[22] Diafas A., Ghadiri N., Beare N., Madhusudhan S., Pearce I., Tan S.Z. (2022). Comment on: “Paracentral acute middle maculopathy and acute macular neuroretinopathy following SARS-CoV-2 infection”. Eye.

[23] Virgo J., Mohamed M. (2020). Paracentral acute middle maculopathy and acute macular neuroretinopathy following SARS-CoV-2 infection. Eye.

[24] Sonmez H.K., Polat O.A., Erkan G. (2021). Inner retinal layer ischemia and vision loss after COVID-19 infection: A case report. Photodiagnosis Photodyn. Ther..

[25] Giacuzzo C., Eandi C.M., Kawasaki A. (2022). Bilateral acute macular neuroretinopathy following COVID-19 infection. Acta Ophthalmol..

[26] Jalink M.B., Bronkhorst I.H.G. (2022). A Sudden Rise of Patients with Acute Macular Neuroretinopathy during the COVID-19 Pandemic. Case Rep. Ophthalmol..

[27] Zamani G., Ataei Azimi S., Aminizadeh A., Shams Abadi E., Kamandi M., Mortazi H., Shariat S., Abrishami M. (2021). Acute macular neuroretinopathy in a patient with acute myeloid leukemia and deceased by COVID-19: A case report. J. Ophthalmic Inflamm. Infect..

[28] David J.A., Fivgas G.D. (2021). Acute macular neuroretinopathy associated with COVID-19 infection. Am. J. Ophthalmol. Case Rep..

[29] Masjedi M., Pourazizi M., Hosseini N.-S. (2021). Acute macular neuroretinopathy as a manifestation of coronavirus disease 2019: A case report. Clin. Case Rep..

[30] Deshmukh R., Raharja A., Rahman F., Petrushkin H. (2021). Optical Coherence Tomography Angiography-Confirmed Paracentral Acute Middle Maculopathy Associated with SARS-COV-2 Infection. J. Neuro-Ophthalmol. Soc..

[31] Zaheer N., Renju M.P., Chavan R. (2022). Acute macular neuroretinopathy after COVID-19 vaccination. Retin. Cases Brief Rep..

[32] Afonso M.G., Marques J.H., Monteiro S., Lume M., Abreu A.C., Maia S. (2021). Acute macular neuroretinopathy following SARS-CoV-2 vaccination. Retin. Cases Brief Rep..

[33] Pichi F., Aljneibi S., Neri P., Hay S., Dackiw C., Ghazi N.G. (2021). Association of Ocular Adverse Events with Inactivated COVID-19 Vaccination in Patients in Abu Dhabi. JAMA Ophthalmol..

[34] Patel S.N., Yonekawa Y. (2022). Acute macular neuroretinopathy after SARS-CoV-2 vaccination. Retin. Cases Brief Rep..

[35] Bøhler A.D., Strøm M.E., Sandvig K.U., Moe M.C., Jørstad Ø.K. (2022). Acute macular neuroretinopathy following COVID-19 vaccination. Eye.

[36] Book B.A.J., Schmidt B., Foerster A.M.H. (2021). Bilateral Acute Macular Neuroretinopathy After Vaccination Against SARS-CoV-2. JAMA Ophthalmol..

[37] Dehghani A., Ghanbari H., Houshang-Jahromi M.-H., Pourazizi M. (2022). Paracentral acute middle maculopathy and COVID-19 vaccination: Causation versus coincidence finding. Clin. Case Rep..

[38] Mambretti M., Huemer J., Torregrossa G., Ullrich M., Findl O., Casalino G. (2021). Acute Macular Neuroretinopathy following Coronavirus Disease 2019 Vaccination. Ocul. Immunol. Inflamm..

[39] Bolletta E., Iannetta D., Mastrofilippo V., De Simone L., Gozzi F., Croci S., Bonacini M., Belloni L., Zerbini A., Adani C. (2021). Uveitis and Other Ocular Complications Following COVID-19 Vaccination. J. Clin. Med..

[40] Ishibashi K., Yatsuka H., Haruta M., Kimoto K., Yoshida S., Kubota T. (2022). Branch Retinal Artery Occlusions, Paracentral Acute Middle Maculopathy and Acute Macular Neuroretinopathy after COVID-19 Vaccinations. Clin. Ophthalmol..

[41] Malerbi F.K., Schoeps V.A., Matos K.T.F. (2022). Paracentral acute middle maculopathy in Susac syndrome after dual exposure to SARS-CoV-2 antigen. BMJ Case Rep..

[42] Sanjay S., Kawali A., Mahendradas P. (2022). Acute macular neuroretinopathy and COVID-19 vaccination. Indian J. Ophthalmol..

[43] Drüke D., Pleyer U., Hoerauf H., Feltgen N., Bemme S. (2021). Acute macular neuroretinopathy (AMN) following COVID-19 vaccination. Am. J. Ophthalmol. Case Rep..

[44] Valenzuela D.A., Groth S., Taubenslag K.J., Gangaputra S. (2021). Acute macular neuroretinopathy following Pfizer-BioNTech COVID-19 vaccination. Am. J. Ophthalmol. Case Rep..

[45] Rennie A.T., DeWeerd A.J., Martinez M.G., Kay C.N. (2022). Acute Macular Neuroretinopathy Following COVID-19 mRNA Vaccination. Cureus.

[46] Bellur S., Zeleny A., Patronas M., Jiramongkolchai K., Kodati S. (2022). Bilateral Acute Macular Neuroretinopathy after COVID-19 Vaccination and Infection. Ocul. Immunol. Inflamm..

[47] Franchi A., Rauchegger T., Palme C., Frede K., Haas G., Blatsios G., Kralinger M., Zehetner C. (2022). Two Cases of Acute Macular Neuroretinopathy Associated with the Adenovirus-based COVID-19 Vaccine Vaxzevria (Astrazeneca). Ocul. Immunol. Inflamm..

[48] Gabrielle P.-H., Baudin F., Ben Ghezala I., Meillon C., Bron A.M., Arnould L., Creuzot-Garcher C. (2022). Bilateral acute macular neuroretinopathy in a young woman after the first dose of Oxford-AstraZeneca COVID-19 vaccine. Am. J. Ophthalmol. Case Rep..

[49] Chen S., Hodge C. (2022). Comment on: “Acute macular neuroretinopathy following COVID-19 vaccination”. Eye.

